# COVID-19 changes to the pregnancy and birth assistance: Catalan midwives’ experience

**DOI:** 10.18332/ejm/138705

**Published:** 2021-07-12

**Authors:** Pablo Rodríguez Coll, Eva Gilaberte Martínez, Falip Dolores Roca, Ramón Escuriet Peiró

**Affiliations:** 1 GHenderS Research Group, Blanquerna School of Health Science, University Ramon Llull, Barcelona, Spain; 2 Department of Obstetrics and Gynecology, Germans Trias and Pujol Hospital, Barcelona, Spain; 3 Health and Integrated Care division, Catalan Health Service, Barcelona, Spain

**Keywords:** antepartum care, childbirth education, intrapartum care, midwifery professional issues, public health

## **Dear Editor**,

At the beginning of January 2020 in Wuhan (China), a new and highly contagious RNA virus (SARS-CoV-2) was identified by the Chinese authorities^1^. This virus caused the COVID-19 disease, which may be asymptomatic or have respiratory or gastrointestinal symptoms that range from mild to severe. Two months later, the World Health Organization (WHO) declared COVID-19 as a global pandemic pointing to the almost 120000 cases of coronavirus illness in more than a hundred countries around the world.

The first wave of the COVID-19 pandemic in Spain changed drastically our sanitary healthcare outlook and specifically midwives’ work. Firstly, many maternity hospitals had to transfer their women and families to third-level hospitals because they were on the verge of collapsing. Secondly, hundreds of midwives were assigned to different services since their delivery rooms were closed, whereas other maternity hospitals saw a significant increase in their workload with a considerable lack of resources and have even restricted partner’s presence for the women in labor.

Thirdly, in healthcare facilities, there was a significant reduction in the number of face-to-face consultations to prevent the spread of the virus and decrease people’s movement and gathering.

Despite the decrease in the birth rates by 4.2% in the first half of 2020^2^, in the area of healthcare belonging to the north Barcelona region, the Germans Trias and Pujol Hospital had increased by 10% the number of births attended due to the closing of the Espiritu Santo birth Hospital^3^. Another important aspect to take into account is the increase in the number of coordinated hospital discharges of postpartum women (between 24 and 32 hours) with primary healthcare by 366% (from 89 in 2019 to 415 in 2020)^3^. This program started in 2017 and was created to ensure the continuity of care, where the postpartum women are visited by the community midwives at home providing quality, holistic, family-centered care.

Regarding our hospital breastfeeding rates, we observed an increase of 21.6% in exclusive breastfeeding. However, there is also an increase of 6.33% in the formula feeding willingness (from 4.2% in 2019 to 10.5% in 2020). Furthermore, the breastfeeding drop-out rate during the hospital stay was 2.6%.

Therefore, the pandemic dramatically changed professionals’ and women’s minds regarding pregnancy and birth assistance. However, little is known about midwives’ experience in providing care during the pandemic in Spain so far^4^. The recently empowered women and families have led to a confusing paradigm where they turn to their self-learning in pregnancy and breastfeeding aspects due to the midwife’s support absence during pregnancy and birth, contrary to the individualized ‘one-to-one care’ established by the World Health Organization Intrapartum Care Initiative^5^ and the Care Strategy for Normal Childbirth^6^. In our healthcare referral area, not only midwives have experienced professional and personal challenges during the pandemic related to remote consultation training and strict infection control equipment and procedures when attending women and their families, but also have experienced the lack of breastfeeding counseling and emotional support to women and families attended, increased the use of formula-feeding and early breastfeeding retirement. Furthermore, midwives refer to missed opportunities to identify important issues such as domestic violence, mental health problems, or women with language problems in the virtual antenatal visits.

Policymakers and organizations should consider lessons learned to support the fact that midwives are a valuable resource that needs enough human staff, equipment and support to provide safe and respectful, holistic and family-centered care to women and their families in future health crises.
